# Surface Free Energy Determination of APEX Photosensitive Glass

**DOI:** 10.3390/mi7030034

**Published:** 2016-02-23

**Authors:** William R. Gaillard, Emanuel Waddell, John D. Williams

**Affiliations:** 1 Department of Electrical and Computer Engineering, University of Alabama in Huntsville, Huntsville, AL 35899, USA; wrg0001@uah.edu; 2 Department of Chemistry, University of Alabama in Huntsville, Huntsville, AL 35899, USA; emanuel.waddell@uah.edu

**Keywords:** surface free energy, surface tension, contact angle, photosensitive glass, microfluidics

## Abstract

Surface free energy (SFE) plays an important role in microfluidic device operation. Photosensitive glasses such as APEX offer numerous advantages over traditional glasses for microfluidics, yet the SFE for APEX has not been previously reported. We calculate SFE with the Owens/Wendt geometric method by using contact angles measured with the Sessile drop technique. While the total SFE for APEX is found to be similar to traditional microstructurable glasses, the polar component is lower, which is likely attributable to composition. The SFE was modified at each stage of device fabrication, but the SFE of the stock and fully processed glass was found to be approximately the same at a value of 51 mJ·m^−2^. APEX exhibited inconsistent wetting behavior attributable to an inhomogeneous surface chemical composition. Means to produce more consistent wetting of photosensitive glass for microfluidic applications are discussed.

## 1. Introduction

The thermal, electrical, and chemical properties of photosenstive glass make it well suited for radio frequency (RF) and integrated circuit (IC) packaging, optoelectronics, micro-optics, microfluidics, and optofluidics. Perhaps the greatest commercial and research interest for photosensitive glass technology is in micro- and optofluidics. Microfluidic systems are designed to transport, dispense, combine, separate, detect, and/or characterize fluid samples. Integration of these processes into a single micro-device when sample volumes are small is the primary advantage achieved through miniaturization. In order to obtain and optimize control of fluids at the microscale, governing forces such as electrostatics, van der Waals forces, and surface energy must be well understood. In this effort the latter force parameter is studied by measuring contact angles of several test liquids on photosensitive glass. Less is known about photosensitive, also termed photodefinable and photostructurable, glasses despite offering many advantages over traditional microstructurable glasses for microfluidics [1,2,3,4]. As a relatively new product, many features and processing aspects of APEX have not been characterized. Surface energy is one such parameter that has yet to be studied. The determination of surface energy of photosensitive glass has the greatest impact in microfluidics since device operation is dependent on solid-liquid interactions.

Several terms are used to describe the surface energy of phase boundaries. The work required to modify the size of an interface between adjacent phases is known as interfacial tension. If the adjacent phases are liquid/gaseous or solid/gaseous, the interfacial tensions are referred to as surface tension and surface free energy (SFE), respectively. The SFE of a substrate will determine the surface tension of a liquid on the surface. Surface tension in turn predicts wettability, dispersibility, and droplet size and formation.

In microfluidics, SFE and surface tension have wide influence on device operation and design. As systems scale down, the surface-to-bulk ratio of fluids becomes larger, and surface tension increasingly dominates over other forces such as gravity or pressure. In microelectromechanical systems (MEMS), surface tension is often a problem to be overcome [5,6], and while this remains true in microfluidics, it can be a useful or even critical design feature [7]. For example, pumps and valves actuated by inflating and deflating gas bubbles rely on surface tension for the formation, stability, and localization of bubbles [8,9,10]. Fluid transport can be achieved via surface tension gradients generated by Marangoni, continuous electrowetting, or passive capillary effects [11,12,13]. Applications utilizing surface tension gradients range from the self-filling of microchannels to sample collection and microscale propulsion. Furthermore, surface tension is factored into designs of pumps, traps, filters, and switches [11,14,15,16,17]. Surface tension–confined microfluidics relies on sharp differences and gradients to two-dimensionally confine and manipulate fluids [16]. Capillarity, wetting, and adhesion are all dependent on surface tension. These properties govern channel filling/filling rate, capillary rise height/rise time, and bubble and droplet formation/size/motion [14,15]. All of these surface tension and SFE effects allow designers of microfluidic experiments and devices to optimally control fluids in the micro-environment.

Additionally, surface wettability and roughness have a direct impact on flow velocity. Boundary slip occurs more easily between fluids and hydrophobic surfaces due to decreased flow resistance compared to hydrophilic surfaces [18,19]. Boundary slip not only results in higher average velocities in hydrophobic microchannels, but as flow rate increases the gap between average flow velocity in hydrophobic and hydrophilic microchannels also increases [20]. Roughness modifies flow velocity depending on surface state. Increased roughness of hydrophobic surfaces inhibits boundary slip which reduces flow velocity [21,22]. For hydrophilic surfaces, increased roughness decreases flow velocity, but the effect is attributable to increased friction resistance [20].

For channel depths less than 100 µm, high quality microstructured surfaces are routinely produced in traditional microfluidic glasses with wet etching or laser ablation [23,24,25]. A high surface roughness for deeper channels occurs with laser ablation due to redepositon of ablated glass [26,27]. Defects in the sacrificial mask layer, stresses in the mask or glass, and redeposition of insoluble products increase surface roughness of wet-etched channels. While methods have been developed to mitigate these sources of defects, the channel profiles that result are circular or trapezoidal [23,28]. Microstructures are produced in photosensitive glass without the use of a sacrificial mask layer, which eliminates many defects and simplifies the fabrication process. High aspect ratios, and transparent and nearly rectangular profiles have been demonstrated in Foturan and APEX photosensitive glasses [29,30,31,32,33]. These advantages allow micro-optical components to be embedded directly into glass lab-on-a-chip devices.

In this study, five samples of APEX glass were prepared at different production stages of a complete microfluidic device. By measuring the SFE at each step of device fabrication, the impact individual steps have on the final SFE can be evaluated. To determine SFE, contact angles of distilled (DI) water, formamide, ethylene glycol, and bromonaphthalene on all five samples were measured with the Sessile drop technique. The Owens/Wendt geometric mean method was implemented to calculate SFE from contact angle data [34]. Additionally, the surface roughness of each sample was measured with a white light interferometer. Surface morphology indicates local compositional variance and is thought to be the primary factor contributing to SFE disparities between the APEX samples. The SFE of APEX is compared to two studies of soda-lime glass from the literature. Chemical composition is the likely explanation for a more dispersive and less polar SFE for APEX compared to soda-lime glass.

## 2. Experimental Procedure

### 2.1. Glass Processing

It is difficult to characterize the SFE of microfluidic channels directly. Therefore, large area samples were prepared to produce a surface similar to a microchannel. A total of five 1.5 cm × 3.0 cm substrates were diced from a half-millimeter-thick wafer. The diced glass was then rubbed with ethanol, thoroughly rinsed with ethanol, and dried under a stream of nitrogen. Each sample was processed to a different fabrication stage in the production of a microfluidic device.

At the first stage a pattern is transferred from a photomask to the glass by exposure to a 7 joule dose of 280–310 nm light. A latent image of the photomask pattern is revealed in the exposed samples after a two-step baking procedure [32]. For the first bake step, the glass is sandwiched between polished alumina substrates and heated from room temperature to 500 °C at a rate of 6 °C/min. The temperature is held constant for 75 min before initiating the second step by ramping up the temperature to 555 °C at a rate of 2 °C/min. The oven temperature is then kept at 555 °C for 80 min before ramping down to room temperature at a rate of 2 °C/min. Baking completes a photothermal cycle to produce metal colloids that nucleate to form a crystalline microphase revealing the pattern transferred through ultraviolet (UV) illumination. After 1 h in 10% hydrofluoric acid (HF), the patterned glass is etched completely through, while the remaining glass is reduced from a thickness of 500 µm to approximately 400 µm. For this preparation, samples were etched for 30 min.

To reduce the surface roughness produced by etching, a final anneal stage is implemented. The anneal consists of a 6 °C/min ramp from room temperature to 535 °C, and 7 h dwell before ramping back to room temperature at 2 °C/min. Table 1 summarizes the processing steps for each sample. Sample 4 represents a surface that is most similar to a microfluidic channel or reactor wall. Since patterned regions are completely removed after etching, no sample was exposed to UV light. Annealing bonds the base, microchannel, and via layers to produce a microfluidic device. Channels of a desired depth are formed by over etching.

### 2.2. Contact Angle Measurement

After fabrication and between-contact-angle measurements, glass surfaces were prepared by rubbing with ethanol and shed resistant cleanroom wipes to remove any residue that could not be removed by rinsing. The glass was then thoroughly rinsed in DI water, immersed in 5% nitric acid for 15 min, and dried under a stream of nitrogen. Cleaning procedures vary, but a final step before measuring contact angles on glass substrates is often to outgas the substrates to remove physisorbed water [35,36]. Outgassing at over 100 °C for several hours reduces the contact angle of water on glass from 30° to 40° to approximately zero [37,38,39]. The SFE for glass with physisorbed water will have a higher polar component than glass with the surface water removed. For microfluidic applications outside of a lab, heating a device to a high temperature for several hours before use is impractical. For this reason, outgassing of the APEX samples was not performed. Thus, the SFE reported here is a practical working value for uncoated glass as opposed to the true native SFE.

A Ramé-Hart model 290 F4 series automated goniometer (Ramé-Hart Instrument Co., Succasunna, NJ, USA) was used to obtain contact angle measurements. Samples were placed under a stainless steel needle with 0.14 mm inner diameter. Droplets measuring 4 µL were dispensed onto glass samples by the touch off method. The goniometer apparatus includes a camera and light source to capture droplet images, and DROPimage software (Ramé-Hart Instrument Co.) for automatic calculation of contact angles. Prior to APEX measurements, contact angles of DI water on a Teflon standard were evaluated to ensure proper goniometer operation. Measurements were carried out at room temperature without controlling for humidity.

The selected test liquids were DI water, ethylene glycol, formamide, and bromonaphthalene. The dispersive and polar components of these test liquids are provided in Table 2 and are used for the geometric calculation of SFE. A total of six droplets for each test liquid were dispensed onto each APEX sample. Drops were dispensed only after the previous drop was measured. Droplets were allowed to equilibrate for several seconds before measurements were taken. The mean contact angle of each drop was measured 6 times in rapid succession and averaged to produce a single data point. This procedure was repeated twice; therefore, a total of 18 droplets for each test liquid provided 72 data points on each APEX substrate. Glassware used to handle the test liquids was cleaned with detergent, thoroughly rinsed with DI water, and dried on a hotplate.

### 2.3. Surface Roughness Measurement

Surface roughness measurements were obtained with a Wyko NT 1100 white light interferometer (Veeco, Tucson, AZ, USA). Before measuring, glass samples were cleaned with the same procedure as described for contact angle measurements. High magnification vertical scanning interferometry (VSI) with a 20× objective and 2× field of view was implemented for each measurement. Six randomly selected scans of 146 µm × 111 µm were imaged onto a 736 × 480 pixel charge-coupled device (CCD) for each sample. The developed interfacial area (*S*_dr_) was obtained for each scan with tilt correction but no additional data manipulation. *S*_dr_ is the percentage of the additional surface area of a textured surface to that of an ideal flat plane of the same size. The use of *S*_dr_ has been shown to predict wetting modified by roughness better than other measures of surface roughness, such as the average or root-mean-squared parameters [43,44].

## 3. Results and Discussion

### 3.1. Contact Angle and Surface Roughness

**Figure 1:**
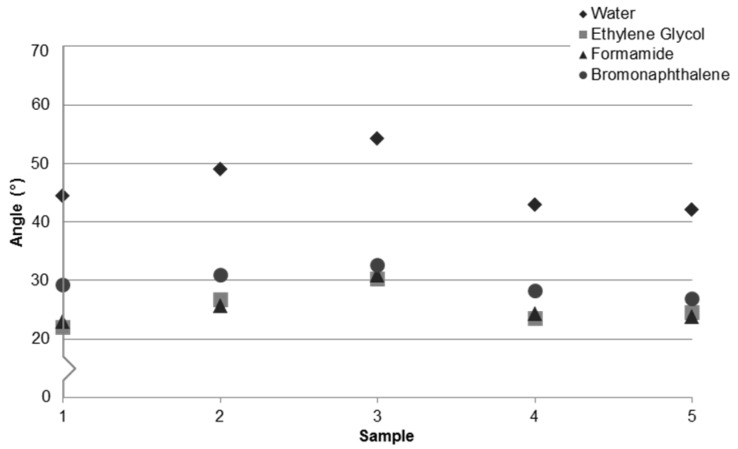
Contact angles on APEX glass samples. Each data point represents the average value of measurements from 18 droplets. Measurement error ranged from ±0.07° to ±1.70° and averaged ±0.37°.

Contact angle measurements of the four test liquids from Table 2 on APEX glass samples from Table 1 are provided in Figure 1. The difference in contact angle for each test liquid on a particular sample and the difference in wetting for the same test liquid across samples can be understood by considering SFE. The surface energy of liquids and solids is comprised of polar (dipole, Lewis acid-base, *etc.*) and dispersive (Lifshitz-van der Waals) contributions. Wetting behavior depends on both polar and dispersive components of the liquid and solid; however, the polar interactions are more dominant in predicting contact angles for high energy surfaces such as glass [45,46]. Contact angles exhibited by ethylene glycol and formamide are similar, as expected based on their comparable polar and dispersive components. As a purely dispersive liquid, bromonaphthalene shows the least variation across samples. Water with a high polar component exhibits the greatest variation.

When comparing any two samples from Figure 1, a consistent increase or decrease in average contact angle for all test liquids is observed, indicating that the surfaces are dissimilar. It was previously shown that the surface roughness is modified after each processing step [32]. Change in wetting behavior due to surface roughness is well established, so it was thought that the difference in wetting would be consistent with roughness variation between samples. However, as will be shown, the modification to wetting is likely due to surface composition disparities, while surface roughness is a minor contribution.

The range in contact angle values across all samples and liquids in Figure 1 is ±10°–15°. Before considering the influence of surface roughness, contamination and surface state were identified as possible sources for this large variance and were investigated.

Chemical contamination of the substrates or solutes in the test liquids can affect wetting; however, these effects, if present, would be consistent across measurements, leading to both an increase in and a broadening of observed angles [47]. To determine if contamination was the primary factor giving rise to contact angle variance, the samples were rinsed in ethanol after cleaning to coat the substrates with organic residues. This led to an increase in average contact angle for each sample but no change in the relative range of measurements.

In a second test, the samples were heated to 125 °C for 24 h after cleaning to evaporate physisorbed water and contaminants that may have been left after cleaning. This led to a decrease in average contact angle but no change in variance. Therefore, cleanliness is unlikely to be the main contributor to measurement deviations, since the average observed angle could be increased without broadening the data range when contaminants were added. Likewise, the average contact angle could be lowered without narrowing the relative range of measurements after evaporating surface water and potential contaminants. If the sample preparation were inadequate and contamination was the dominate factor in average contact angle range, it is unlikely that the range would be unaffected by substantial changes to the cleaning procedure.

**Figure 2:**
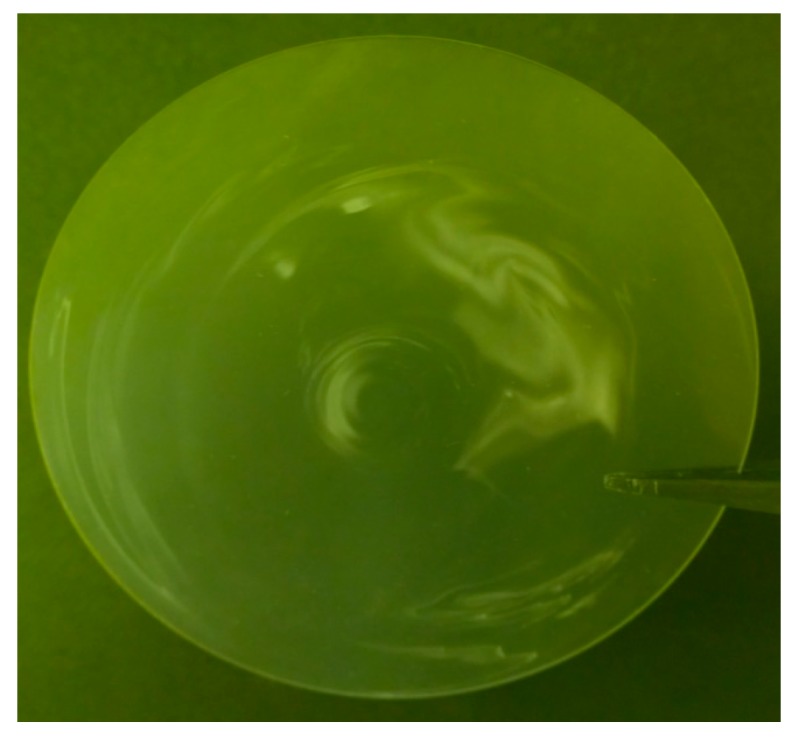
Visible striations in unpatterned four inch diameter wafer after bake and etch processes.

**Figure 3:**
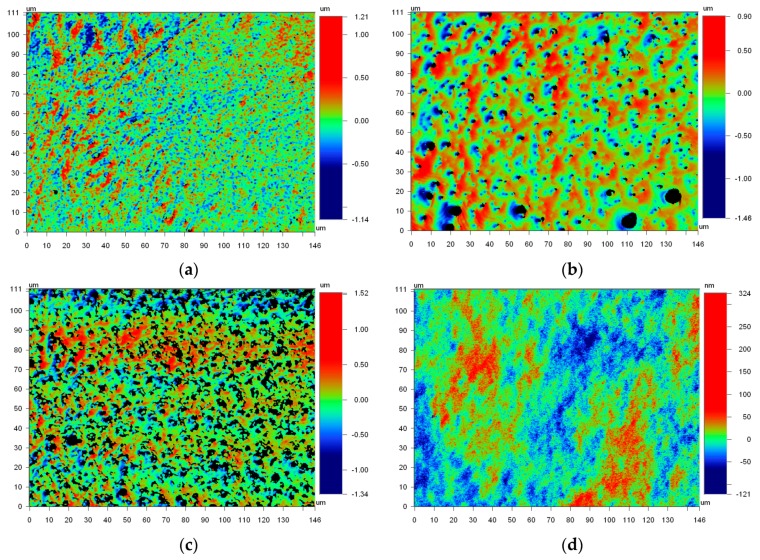
Variation in morphologies of surface striations from (**a**) sample 3, (**b**) sample 4, and (**c**) sample 5; and (**d**) surface morphology of transparent region from sample 4.

Ruling out cleanliness for the large range in contact angles, our attention turned to substrate surface state. An inhomogeneous distribution of glass components is revealed after baking and becomes more distinct after etching. Sufficient heating allows the various constituents to migrate and cluster, forming visible striations as in Figure 2. These striations exhibit distinct surface morphologies that differ substantially from transparent regions as indicated in Figure 3. A wide range of surface textures can result, and several possible morphologies are described in [2]. The striation morphologies vary across individual samples, and more variations exist than are presented in Figure 3. All commercially available photosensitive glasses exhibit similar striation patterns.

We postulated that morphological variance indicates surface composition inhomogeneity over the entire substrate, which gives rise to the observed contact angle range across all samples and test liquids. To mitigate surface state variation, the substrates were silanized in a nitrogen environment for 15 min with a 15 mg·mL^−1^ solution of chlorotrimethylsilane (CTMS) mixed in acetonitrile. The silane coating increased the average contact angle of DI water and reduced the range from ±10°–15° to ±2°–4°. This is strong evidence that the wetting of APEX glass is highly dependent on local surface composition at all processing stages.

Uniform roughness is assumed in mathematical models developed to predict modifications to wetting as a function of surface roughness. The diverse surface textures of Figure 3 indicate surface roughness disparities across individual substrates. However, as reported in Table 3, the surface roughness range on individual samples is low, allowing uniform surface roughness to be assumed for modeling. Future work may validate this assumption through simultaneous measurement of surface roughness and contact angles evaluated at different locations on the same sample.

**Figure 4:**
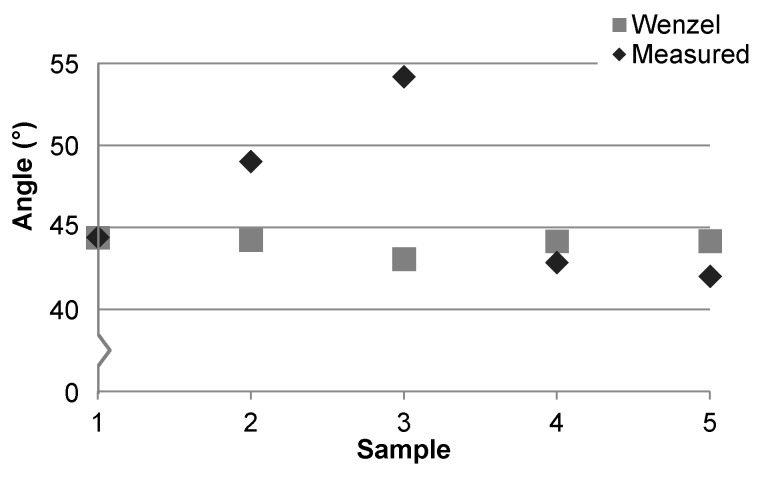
Measured and predicted contact angles of DI water based on *S*_dr_.

The influence of surface roughness on wetting is modeled with the Wenzel, Cassie-Baxter, or hybrid theories. The Cassie-Baxter model was not evaluated as it is typically applied to hydrophobic or rough surfaces. Hybrid models were not considered as they are more complex but offer no better predictability than the Wenzel model when surface roughness is low [43,44]. The *S*_dr_ values in Table 3 were used to predict contact angles for each surface based on the Wenzel theory [43,48]. The measured and predicted contact angle results for DI water on each sample are provided in Figure 4. The trend in measured contact angle values does not match well with the Wenzel model, indicating that roughness alone cannot explain the observed contact angles across samples. Moreover, if surface roughness was the primary factor for wetting differences between samples then the measured contact angles for samples 1 and 2 should be more similar, as they are for samples 4 and 5. The surface roughness, range, and texture observed for samples 1 and 2 are similar to each other and are more similar in appearance to Figure 3d than Figure 3a–c. This is further evidence for non-uniform surface composition as the primary factor driving the discrepancy in both the average and range of contact angle values between samples. The increase in roughness for sample 3 is due to HF etching [32] and is discussed in further detail in Section 3.2.

### 3.2. Surface Free Energy

**Figure 5:**
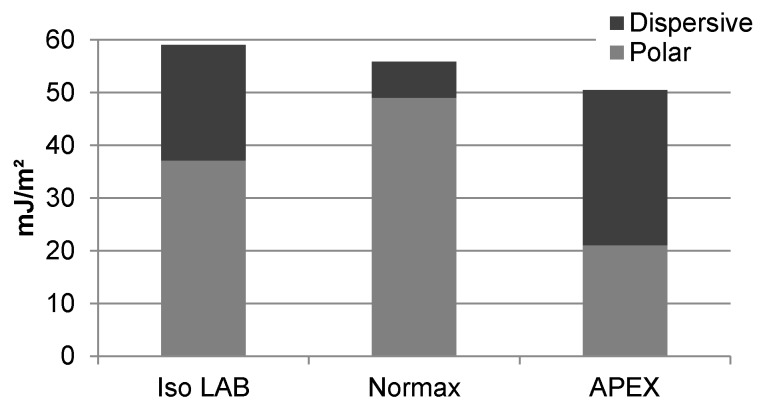
Surface free energy (SFE) of unprocessed APEX glass (sample 1) compared to soda-lime glass microscope slides.

The total SFE of APEX was found to be similar to other glasses in the literature. Figure 5 compares unprocessed APEX (sample 1) to two studies of the SFE for Iso LAB and Normax microscope slides [39,49]. Both microscope slide brands are soda-lime glasses and likely have similar composition, uniformity, and surface roughness. The difference in SFE between the two is attributable to how SFE was determined in each study. Understanding the discrepancy between the two results provides insight into the SFE we report for APEX.

For all three glasses the Owens/Wendt geometric mean method was implemented to calculate SFE. The Iso LAB slides were immersed in water, and contact angles of air bubbles produced at the glass-water interface were measured. The Normax slides were heated to remove physisorbed water, and contact angles were obtained with the Sessile drop technique. The total SFE of the soda-lime glasses is similar as indicated in Figure 5, but the ratio of SFE components is not. Assuming similar surface roughness and composition, the difference in SFE components can be reasonably attributed to the reduction of physisorbed water for the Normax slides. Likewise, a similar total SFE but lower dispersive component for APEX is expected for substrates that are heated. Thus, a lower total SFE and higher dispersive component for APEX compared to the soda-lime glasses is a result of contributions of physisorbed water and differences in oxide additives listed in Table 4.

**Figure 6:**
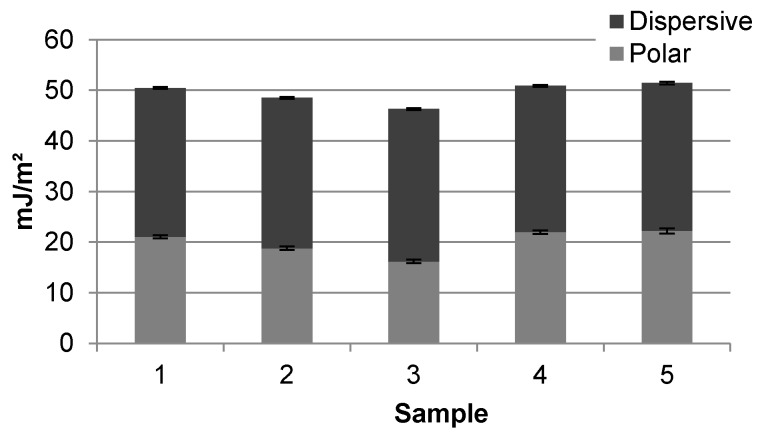
SFE components with error bars of APEX glass samples.

The SFE for each APEX sample was calculated and is presented in Figure 6. The ratio of the polar and dispersive contribution to the total SFE is similar between samples, but the total varies slightly. This difference can be explained by surface composition inhomogeneity as follows. Each additive in the glass will modify the HF etch rate of the silica in the immediate locality to a different degree. If the distribution of constituents within the glass is uneven, then some areas will etch faster than others, giving rise to an increase in surface composition disparity. This is evidenced by the difference in contact angles on samples 1 and 2 despite similar surface roughness. Subsequent etching, as was done for sample 3, further increases surface composition inhomogeneity. Post-pattern baking and annealing initiates migration and agglomeration of similar species within the glass. Samples 1, 4, and 5 have similar wetting behavior and nearly identical SFEs, which indicates that annealing increases homogeneity by reflow and redistribution of surface components. The total SFEs for samples 1 and 4 are 50.5 and 50.9 mJ·m^−2^ with errors of ±0.18 and ±0.23 mJ·m^−2^, respectively. Thus, the SFE and wetting behavior of APEX glass is altered at each fabrication stage, but with regard to wetting the initial and final surface is essentially unmodified by the microfabrication procedure.

## 4. Conclusions

Evaluation of surface roughness and cleanliness leave chemical composition as the most likely explanation for the difference in the relative polar and dispersive contributions to the total SFE of APEX compared to soda-lime glass. X-ray photoelectron spectroscopy (XPS) can verify surface composition inhomogeneity, but XPS analysis is beyond the scope of this work. Simultaneous topography and tensiometer or goniometer analysis can provide a better estimate for the influence of roughness on wetting due to the varied morphology across samples. Still, we have shown that surface roughness resulting from microfabrication has little impact on the wetting of APEX glass compared to the influence of composition inhomogeneity.

The initial surface composition of the stock APEX is likely non-uniform, as evidenced by the significant reduction in contact angle range after application of a silane coating. Compositional inhomogeneity results in a large range in wetting behavior. Of the processing steps, etching of baked glass has the greatest impact on modification to SFE and wetting. When baked, oxide additives form nanoclusters which etch at different rates. Subsequent HF exposure produces a surface with increased roughness, texture, and compositional variance. Annealing returns the surface to a similar state as the stock glass and reduces roughness from preceding microfabrication steps. Adjustments to the fabrication procedure can result in changes to the final overall surface characteristics. However, predictability in local characteristics can only be achieved if the oxide distribution of the stock glass is more uniform.

Surface uniformity is required to achieve precise control in microfluidic applications that rely on SFE for functionality and performance. For photosensitive glass, improvements to the formulation and melting process are needed to achieve a more homogeneous surface. Until such improvements are implemented, a coating to mitigate surface chemical inhomogeneity is recommended for microfluidic applications due to the wide range in wetting behavior on uncoated substrates. One of the advantages of glass for microfluidics is the chemical resistance of glass to a broad range of chemistries. Coating reagents such as CTMS provide a chemically uniform surface while preserving the inertness of the glass. Alternative coating options may be found in [51].

## Figures and Tables

**Table 1:** Processing steps for APEX glass samples.

Sample	1	2	3	4	5
UV	-	-	-	-	-
Bake	-	-	x	x	x
HF	-	x	x	x	-
Anneal	-	-	-	x	x

**Table 2:** Dispersive and polar surface energy components (mN·m^−1^) for selected test liquids from the literature.

Test Liquid	Polar	Dispersive
Distilled Water [40]	50.3	22.5
Ethylene Glycol [41]	16.0	32.8
Formamide [41]	23.5	34.4
Bromonaphthalene [42]	0.0	44.4

**Table 3:** Surface roughness values of APEX samples from Table 1.

Sample	*S*_dr_	Range
1	0.29	±0.06
2	0.26	±0.04
3	2.22	±0.71
4	0.39	±0.10
5	0.36	±0.08

**Table 4:** The wt % of glasses compared in Figure 5. Limits and typical values of soda-lime wt % from [50] and APEX wt % converted from at % in [33]. Soda-lime may contain traces of SO_3_, Se, Co_3_O_4_, Cr_2_O_3_ and MnO_2_ [50].

Component	Soda-Lime (Limits)	Soda-Lime (Typical)	APEX
SiO_2_	58.22–84.15	73.26	58.50
Na_2_O	9.3–15.19	13.81	1.78
CaO	6.55–12.83	8.78	0
MgO	0–3.95	3.86	0
Al_2_O_3_	0–3.33	0.14	16.64
K_2_O	0–2.31	0.03	4.96
Fe_2_O_3_	0–1.57	0.11	0
TiO_2_	0–1.04	0.01	0
Li_2_O and B_2_O_3_	0	0	16.16
ZnO	0	0	1.28
Ag_2_O	0	0	0.20
Sb_2_O_3_	0	0	0.25
Ce_2_O_3_	0	0	0.22
